# Associations between Temperature and Hospital Admissions for Subarachnoid Hemorrhage in Korea

**DOI:** 10.3390/ijerph14040449

**Published:** 2017-04-21

**Authors:** Suji Lee, Matthias Guth

**Affiliations:** 1 Institute of Health and Environment, Seoul National University, Gwanak-gu, Seoul 151-742, Korea; 2 School of Medicine, Technische Universität München, Arcisstraße 21, 80333 Munich, Germany; matthias.guth@tum.de

**Keywords:** subarachnoid hemorrhage, temperature, climate zones, hospital admission, epidemiology, weather

## Abstract

The relationship between temperature and subarachnoid hemorrhage (SAH) is less studied than that between temperature and myocardial infarction or other cardiovascular diseases. This study investigated the association between daily temperature and risk of SAH by analyzing the hospital admission records of 111,316 SAH patients from 2004 to 2012 in Korea. A Poisson regression model was used to examine the association between temperature and daily SAH hospital admissions. To analyze data and identify vulnerable groups, we used the following subgroups: sex, age, insurance type, area (rural or urban), and different climate zones. We confirmed a markedly higher SAH risk only for people of low socioeconomic status in both hot and cold temperatures; the relative risk (RR) in the Medicaid group was significantly increased and ranged from 1.04 to 1.11 for cold temperatures and 1.10 to 1.11 for hot temperatures. For the National Health Insurance group, the RR was increased to 1.02 for the maximum temperature only. The increased risk for SAH was highest in the temperate zone. An increase above the heat threshold temperature and a decrease below the cold threshold temperature were correlated with an increased risk of SAH in susceptible populations and were associated with different lag effects and RRs.

## 1. Introduction

Subarachnoid hemorrhage (SAH) comprises 1–7% of all stroke cases but is a severe type of stroke [1]. Despite its relative rarity, SAH has a large impact because of the relatively young age of onset and poor outcome [1,2,3]; SAH occurs at younger ages than stroke or intracerebral hemorrhage, the fatality rate is 50%, and half of all survivors are left severely disabled. The exact physiological mechanisms responsible for the increases in SAH incidence associated with hot and cold weather are unknown. Many studies have evaluated risk factors for SAH, and the risk factors of age, female sex, hypertension, and tobacco use are supported by strong evidence [4,5]. In addition, genetic predisposition and alcohol and drug abuse are the most commonly discussed risk factors [6,7,8,9,10,11,12,13,14,15,16,17,18]. However, whether or not seasonal or meteorological factors influence SAH is still a matter of debate. Based on a systematic review and meta-analysis, Piters et al. [19] observed that SAH occurs less often in summer than in winter, and most often in January. However, other studies failed to show any seasonal or meteorological associations [20,21,22]. McDonald et al. [22] did not identify any significant monthly or temperature-related effects in the incidence of SAH, and Besoglu et al. [23] also found that neither season nor weather significantly influences the incidence of SAH.

In the past two decades, numerous epidemiological studies have confirmed that short- or long-term exposure to climatic change contributes to increased risk of cardiovascular diseases such as ischemic heart disease, pulmonary heart disease, cardiac arrhythmia, heart failure, ischemic stroke, and myocardial infarction (MI) [9,13,15,18,24]. Although the effects of high or low temperatures on cardiovascular disease have been well-documented, the effects of temperature specifically on SAH have not been investigated as extensively as the effects on robust outcomes such as MI and heat-related illnesses. Significantly less evidence exists on the effects of temperature on SAH, and the results vary widely between studies. Changing temperature, barometric pressure, and relative humidity have been explored by investigators who identified an association between the incidence of SAH and meteorological changes [10,14,16,17,20]. Several reports confirmed an increased risk of SAH in cold weather or winter [14,16,17]. Gill et al. [12] were the first to report a direct relationship between decreased temperature and an increased risk of SAH, which was supported by another study in France [16]. In contrast, Field and Hill [11] examined daily temperature changes caused by the Chinook winds and the incidence of SAH in the cold winter climate of Calgary, Canada and found no relationship. Rué et al. [25] recently analyzed the influence of daily temperature variation on SAH occurrence in a homogeneous population of 236 SAH patients from a single facility in France; no relationship was observed between mean change in temperature and the occurrence of SAH. Finding a susceptible group, such as the elderly or people with a low socioeconomic status, who might be at increased risk of SAH, is essential. However, no studies have compared groups susceptible to both hot and cold temperatures. This is the first study to investigate the association between daily temperature and SAH hospital admissions using nationwide data covering 97% of Korea’s population from 2004 to 2012.

## 2. Materials and Methods

### 2.1. Data Collection

All SAH admission (International Classification of Diseases, Clinical Modification code 430) records were extracted from the 2004–2012 Korea National Health Insurance Corporation, which covers 97% of Korea’s population. We collected data on cause of hospitalization, sex, age, region, and type of insurance coverage. Climate data were obtained from the Korean Meteorological Administration and included daily mean, minimum, and maximum temperatures in addition to daily precipitation, humidity, dew point, sea level pressure, and wind speed for the study time period. Air pollution data including ambient 24-h average concentrations of particulate matter with an aerodynamic diameter less than or equal to 10 µm (PM_10_) and NO_2_ were provided by the National Institute of Environmental Research in Korea. To analyze the effects of high and low temperatures more specifically, we subdivided the districts by the number of heating degree days (HDDs) and cooling degree days (CDDs) observed during the study period. HDDs were calculated as the sum of the daily temperature values below the mean outside temperature of 18 °C, and CDDs were defined as the sum of the daily temperature values above the mean outside temperature of 24 °C [26]. We then grouped the districts into climate zones according to the number of HDDs and CDDs observed. Population data were obtained from the 2004–2012 Korean National Statistical Office and were used to adjust for temporal population trends by region. Data were collected by National Health Insurance (NHI) and ethical approval was provided by the Ethical Review Committee of the Seoul Hospital at Hanyang University. Data were anonymized before they were provided to us.

### 2.2. Data Analysis

We used piecewise regression analysis to find the threshold temperature in the relationship between daily-adjusted hospital admission and temperature values. Details for this analytical method were previously published [15]. We estimated the temperature-SAH association by applying Poisson generalized additive models with nonparametric smoothing functions (splines) to describe nonlinear relationships. Associations were adjusted according to humidity, barometric pressure at sea level, and air pollutants including PM_10_ and NO_2_, which significantly influenced the estimated risk of SAH [13,18,27]. We also controlled for delayed effects using the following average lag structures: 0 day (on the day of the hospital admission), 1 day, 2–3 days, 4–7 days, 8–14 days, 15–21 days, and 22–28 days. Public holidays were controlled for by use of dummy variables. Data analysis was performed using SAS version 9.3 (SAS Institute Inc., Cary, NC, USA).

## 3. Results

**Figure 1:**
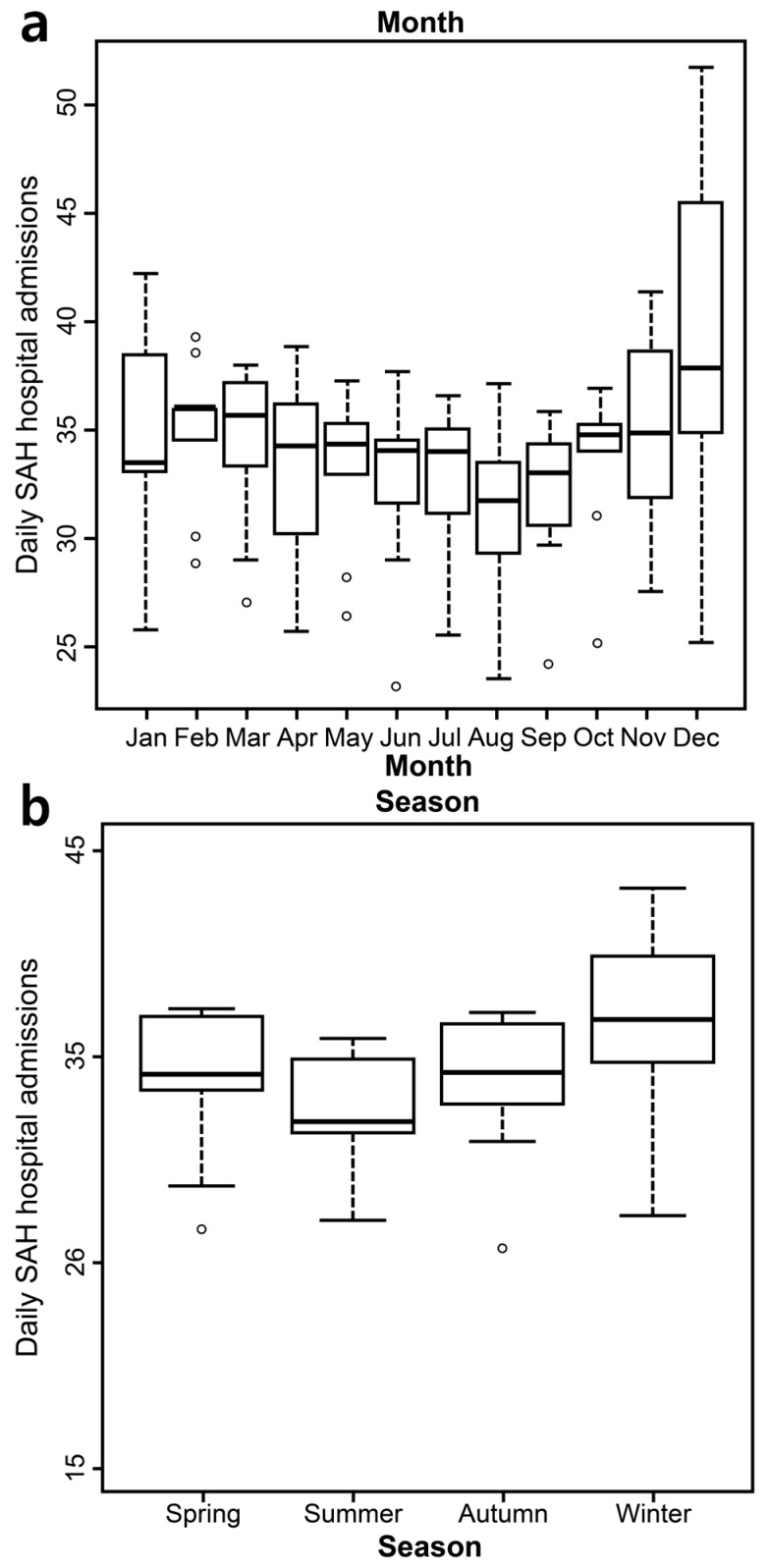
Boxplots showing the daily subarachnoid hemorrhage hospital admissions by (**a**) month and (**b**) season (2004–2012).

During the nine-year study period, the total number of hospital admissions for SAH was 111,316 from 1578 hospitals in Korea. Patients were more likely to be female (62.5%), younger than 75 years old (89.3%), and in the NHI group (92.0%). Most admissions occurred in winter (December, January, and February), but the differences between seasons were not significant (Table 1 and Figure 1). Most SAH admissions occurred in December. However, similar to the seasonal results, the monthly differences were not significant (Figure 1a,b). We subdivided each zone by the number of HDDs and CDDs observed during the study period. The summary statistics of temperature variation by season and climate zone are described in the supplementary information. Compared to the temperate zone, the hot zone had higher mean, maximum, and minimum temperatures in addition to twice as many hot days. The number of cold days and the overall cold duration was higher in the cold zone than in the temperate zone (Tables S1–S3).

**Figure 2:**
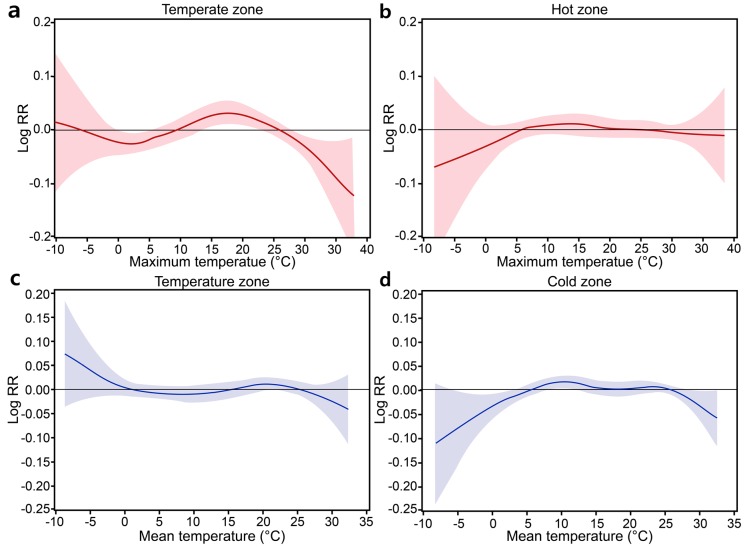
Relationship between hot temperature and subarachnoid hemorrhage (SAH) hospital admissions in temperate and hot zones from 2004 to 2014. Relationships between cold temperature and SAH hospital admissions in temperate and hot zones from 2004 to 2014. (**a**) relationship between hot temperature and SAH in temperate zone; (**b**)relationship between hot temperature and SAH in hot zone; (**c**) relationship between cold temperature and SAH in temperate zone; (**d**) relationship between cold temperature and SAH in cold zone.

The relative risk (RR) of SAH per 1 °C change in the cold mean temperature was higher in the <75 age group (RR = 1.02) and urban group (RR = 1.02). The risk value was higher for the minimum temperature and ranged from 1.02 to 1.09. The RR of SAH per 1 °C change in the cold minimum temperature was higher in the <75 age group (RR = 1.03), males (RR = 1.09), and urban group (RR = 1.05). The RR of SAH per 1 °C change in the hot temperature was not significant in the study population. However, a significantly increased risk of SAH at high temperatures above the mean temperature was found for both the rural (RR = 1.02 at lag of 15–21 days) and urban (RR = 1.02 at lag of 22–28 days) groups as well as the ≥75 age group (RR = 1.05 at lag of 1 day). The RR estimates were higher when analyzing increases in the maximum temperature. The highest estimates were obtained in the ≥75 age group (RR = 1.07 at a lag of 22–28 days) and in rural areas (RR = 1.04 at lag of 8–14 days) (Table 2). For analysis of specific insurance types, we re-estimated the thresholds for cold and hot temperatures because of the relatively low number of cases in the Medicaid group. The results from the NHI group were not significant, except for an increased RR (1.02) at a lag of 22–28 days associated with an above-threshold increase in the maximum temperature. However, the Medicaid group showed significant RRs ranging from 1.04 to 1.11 for cold temperatures and 1.10 to 1.11 for hot temperatures (Table 3). As shown in Table 4, the risk of SAH was significantly increased in the temperate zone in both the cold temperature (HDD) and hot temperature (CDD) analyses. The RR of SAH per 1 °C change in the temperate zone was 1.22 for a cold period and 1.08 for a hot period. Figure 2 illustrates the relationships between temperature and SAH hospital admissions. The risk of SAH increased when the temperature was hot or cold in the temperate zone. However, in the hot and cold zones, the relationship between temperature and the RR of hospital admission presented a flattened pattern, which indicated that temperature was not related to SAH.

## 4. Discussion

The relationship between temperature and risk of SAH is not as well established as that between temperature and MI or other cardiovascular diseases [9,13,15,18,28,29]. Few studies have examined temperature-associated SAH, and the results of previous studies examining associations between weather and SAH incidence are inconsistent [20,21]. For example, Jkovljević et al. [14] observed a significantly increased incidence of ischemic stroke and intracerebral hemorrhage during the winter in Finland; however, the occurrence of SAH did not vary significantly by season there. Lai et al. [30] reported that although the occurrence of SAH was higher during winter and spring compared to other seasons, no seasonal or monthly variations in SAH hospital admissions were evident. We also found no seasonal or monthly variations in SAH hospital admissions, but rather estimated the nonlinear temperature–SAH relationships in both in cold and hot temperatures.

Most previous studies focused on the impact of cold-related health problems on SAH. For example, Piters et al. [19] found a higher occurrence of SAH in January than in the summer months of June–September. In a retrospective study of SAH admissions in the Mid-Atlantic region of the United States, Gill et al. [12] found a statistically significant increase in SAH when a decrease in temperature occurred within a 24-h period. Similarly, Abe et al. [31] confirmed that low temperature may be a risk factor for SAH in Tokyo. Most recently, Zheng et al. [32] reported that the incidence of SAH increased during days with lower temperatures. As expected, we found an association between low temperature and the occurrence of SAH in males, the youngest age group, and urban residents. In addition, the increase in risk associated with cold temperatures occurred with very short time delays. Our results indicated that low temperature over periods of 2–3 days or 4–7 days before onset is a risk factor for SAH (females, lag of 15–21 days).

Our study is the first to report a direct relationship (on the timescale of days) in some vulnerable groups between temperature increase and an increased risk of SAH during hot periods. Few studies have reported a positive correlation between the hot season and incidence of SAH. Kozak et al. [33] observed a peak in the seasonal fluctuation of SAH in the spring through summer. In addition, Landers et al. [34] reported that most patients presented with SAH (46%) between August and November, i.e., during the spring in the southern hemisphere, where the study took place. In South Florida, Tarnoki et al. [35] reported that the increased incidence of SAH cases was consistent with higher air temperature (RR = 0.98, *p* < 0.001). We found that the highest increased risk for SAH was associated with heat exposure, especially in the old age group and among rural residents with more than 8–14 lag d. In contrast to the cold temperatures, hot temperatures showed more delayed effects.

The differences in lag days between the hot and cold effects may be related to the physiological mechanisms of SAH, although the exact physiological mechanisms responsible for the increases in SAH incidence associated with hot and cold weather are unknown. However, lower temperatures can lead to peripheral vasoconstriction, the centralization of circulation, and consequential increases in blood pressure [36]. High temperatures are associated with increased plasma viscosity, blood cell counts, and plasma cholesterol levels [37]. Temperature could also affect the coagulation system; fibrinogen levels and mean platelet volume vary seasonally along with core body temperature [38]. In addition, the temperature and inflammatory response of the human body appear to be associated, as increased levels of inflammatory markers can be found at low outside temperatures [39]. These alterations may explain the increased SAH incidence at higher and lower temperatures.

We identified vulnerable groups in specific analyses (insurance type, climate zone, urban vs. rural). In particular, we confirmed a markedly higher risk for people of low socioeconomic status in both hot and cold temperatures. Socioeconomic status could affect the susceptibility to high and low outdoor temperatures based on the inability or unavailability of technology to control indoor temperature. According to the Centers for Disease Control and Prevention [40], the socioeconomic factors potentially influencing public health include poor housing quality, absence of air conditioning, lack of access to social and health services, and individual behaviors (e.g., alcohol consumption and taking medication). These factors could further increase the susceptibility of these individuals to temperature changes. Such explanations are speculative, as little is generally known about how socioeconomic factors influence the effect of temperature on diseases such as acute MI. However, a lower socioeconomic status has been found to enhance the relationship between temperature and mortality from MI [29]. Moreover, Clark et al. [41] reported an association between low socioeconomic status and large increases in cardiovascular disease risk in men and women in high-income countries.

We found that the RR of SAH was significantly increased by heat exposure in both urban and rural areas. Urban areas showed increased risk more immediately (on the current day) than rural areas. A significantly increased risk for SAH associated with cold temperature was found only in urban areas (RR = 1.05). This may partially be explained by the frequency of farming as an occupation in rural areas of Korea. As farming activity is strongly reduced in winter, the rural population mostly stays inside. This reduced intensity of outside activity could explain why no significant effects of cold temperature were found in rural areas.

We found stronger effects in zones having a generally temperate climate (defined by a low number of HDDs/CDDs). This result is consistent with the findings of studies from other areas worldwide. McDonald et al. [22] divided the North American continent and Hawaii into 11 regions based on 10 °F intervals of the average annual minimum temperatures. They reported that relative to zone 3 (−30 °F through −40 °F), the frequency of SAH admissions was significantly higher in zone 7 (10 °F through 0 °F); these differences amounted to a 20% difference in SAH incidence. In multi-city studies, the most significant effects of cold temperatures were observed in warm regions. The authors hypothesize that people may acclimatize to low temperatures and therefore take more effective adaptive measures against the cold. In contrast, residents of warm regions have fewer physical, social, and behavioral adaptations to low temperatures [42,43]. Braga et al. [44] reported that inhabitants of hot cities are more adapted to high temperatures, probably because of physiologic acclimatization and the high penetration of air conditioning in those places.

## 5. Conclusions

To our knowledge, this is the first study to report a direct relationship between temperature and an increased risk of SAH in both cold and hot periods. Moreover, the results were obtained from a large sample from a national database. This study indicates that hot and cold temperatures strongly contribute to SAH hospital admissions, but no seasonal and monthly variations were evident. The strongest associations were observed between cold temperature and SAH admissions in the groups comprising individuals under 75 years old, females, and urban residents. We also observed the strongest associations between hot temperature and SAH admissions in the groups comprising individuals over 75 years old, males, and rural residents. In particular, people living in a hot or cold climate zone and having a high socioeconomic status were affected less by climatic conditions than those in a temperate climate zone and having a low socioeconomic status.

Our study has several limitations. The first is that our data is from an administrative rather than a medical database. Although this facilitated analysis of an extensive national dataset, our data were coarse and contained no additional medical details for each case. For example, no information was available on the subtype (e.g., ruptured aneurysm or other causes such as arteriovenous malformation) or severity of SAH, or the patients’ past medical history or outcome and whether the SAH was confirmed by computed tomography in each of the cases. In addition, our incidence counts represented the day of hospital admission, not the day of the actual medical event.

Despite these limitations, our findings may have important implications for public health strategies for treating chronic diseases such as SAH in the context of climate change.

## Figures and Tables

**Table 1:** Summary statistics of hospital admissions, temperatures, and air pollutants by season in 2004–2012.

Variable	Spring	Summer	Autumn	Winter	*p*-Value
Hospital admission					
Total SAH	27,891	26,613	27,490	29,322	0.2246
Sex					
Male, % (*n*)	37.88 (10,566)	37.59 (10,004)	37.36 (10,271)	37.00 (10,849)	<0.001
Female, % (*n*)	62.12 (17,325)	62.41 (16,609)	62.64 (17,219)	63.00 (18,473)	
Age					
<75 years, % (*n*)	89.03 (24,830)	89.45 (23,804)	89.42 (24,581)	89.51 (26,247)	<0.001
≥75 years, % (*n*)	10.97 (3061)	10.55 (2809)	10.58 (2909)	10.49 (3075)	
Insurance type					
Medicaid, % (*n*)	7.72 (2154)	8.55 (2276)	7.80 (2144)	7.86 (2305)	<0.001
NHI, % (*n*)	92.28 (25,737)	91.45 (24,337)	92.20 (25,346)	92.14 (27,017)	
Area					
Urban ^a^, % (*n*)	66.89 (18,657)	67.93 (18,079)	67.95 (18,680)	67.59 (19,820)	<0.001
Rural ^b^, % (*n*)	33.11 (9234)	32.07 (8534)	32.05 (8810)	32.41 (9502)	
Temperature zone					
Temperate ^c^, % (*n*)	21.73 (6060)	21.34 (5679)	21.85 (6006)	21.08 (6181)	0.25
Hot ^d^, % (*n*)	22.80 (6359)	23.08 (6143)	23.06 (6338)	23.86 (6997)	
Temperate ^e^, % (*n*)	30.73 (8570)	31.42 (8361)	30.24 (8313)	31.10 (9120)	<0.001
Cold ^f^, % (*n*)	44.16 (12,316)	43.69 (11,626)	44.43 (12,215)	43.65 (12,800)	
Temperature					
Mean (°C)	12.15 (5.81)	24.32 (2.84)	15.28 (6.32)	0.75 (4.71)	
Minimum (°C)	7.23 (5.92)	20.94 (3.1)	10.97 (6.9)	−3.47 (5.05)	
Maximum (°C)	17.66 (6.47)	28.59 (3.47)	20.47 (6.33)	5.58 (5.05)	
Air pollutant					
PM_10_ (µg/m^3^)	64.72 (45.4)	42.69 (22.01)	46.85 (25.25)	56.48 (28.14)	
NO_2_ (ppm)	0.03 (0.01)	0.02 (0.01)	0.03 (0.01)	0.03 (0.01)	

Note: SAH, subarachnoid hemorrhage; SD, standard deviation; Medicaid, medical care; NHI, National Health Insurance; PM_10_, particulate matter with an aerodynamic diameter less than or equal to 10 µm; ^a^ Urban areas with more than 500,000 officially registered in the population; ^b^ Rural areas with fewer than 500,000 officially registered in the population; ^c^ Warm areas with fewer cooling degree days (CDDs); ^d^ Hot areas with more cooling degree days (CDDs); ^e^ Warm areas with fewer heating degree days (HDDs); ^f^ Cold areas with more heating degree days (HDDs).

**Table 2:** Relative risk of subarachnoid hemorrhage with every 1 °C change by subgroup.

Variable	Cold Effect				Heat Effect			
	Mean Temperature (Threshold: −3.5 °C)		Minimum Temperature (Threshold: −13.5 °C)		Mean Temperature (Threshold: 24.5 °C)		Maximum Temperature (Threshold: 31.5 °C)	
	Lag	RR (95% CI)	Lag	RR (95% CI)	Lag	RR (95% CI)	Lag	RR (95% CI)
Total SAH	2–3	1.02 (1.01, 1.03) *	4–7	1.03 (1.00, 1.06) *	15–21	1.01 (0.999, 1.02)	0	1.02 (0.98, 1.06)
Age								
<75 years	2–3	1.02 (1.01, 1.03) *	4–7	1.03 (1.00, 1.06) *	2–3	1.01 (0.99, 1.02)	0	1.04 (0.99, 1.08)
≥75 years	2–3	1.01 (0.99, 1.04)	2–3	1.01 (0.93, 1.11)	1	1.05 (1.00, 1.10) *	22–28	1.07 (1.02, 1.12) *
Sex								
Male	2–3	1.02 (0.999, 1.03)	4–7	1.09 (1.05, 1.14) *	1	1.01 (0.98, 1.03)	22–28	1.03 (1.00, 1.05) *
Female	2–3	1.02 (1.00, 1.03) *	15–21	1.07 (1.03, 1.11) *	2–3	1.01 (0.99, 1.03)	0	1.02 (0.97, 1.07)
Area								
Urban ^a^	2–3	1.02 (1.01, 1.03) *	2–3	1.05 (1.00, 1.09) *	15–21	1.02 (1.01, 1.04) *	22–28	1.02 (1.00, 1.03) *
Rural ^b^	1	1.01 (0.99, 1.03)	4–7	1.02 (0.99, 1.06)	22–28	1.02 (1.00, 1.04) *	8–14	1.04 (1.01, 1.08) *

Note: * *p* < 0.05; SAH, subarachnoid hemorrhage; CI, confidence interval; ^a^ Urban areas with more than 500,000 officially registered in the population; ^b^ Rural areas with fewer than 500,000 officially registered in the population; RR: relative risk.

**Table 3:** Relative risk of subarachnoid hemorrhage with every 1 °C change by insurance type.

Insurance Type	Cold Effect				Heat Effect			
	Mean Temperature (Threshold: −2.5 °C)		Minimum Temperature (Threshold: −12 °C)		Mean Temperature (Threshold: 24.5 °C)		Maximum Temperature (Threshold: 31.5 °C)	
	Lag	RR (95% CI)	Lag	RR (95% CI)	Lag	RR (95% CI)	Lag	RR (95% CI)
Medicaid	2–3	1.04 (1.00, 1.07) *	15–21	1.11 (1.01, 1.19) *	1	1.10 (1.04, 1.16) *	1	1.11 (1.04, 1.19) *
NHI	0	1.01 (0.99, 1.02)	4–7	1.01 (0.99, 1.03)	2–3	1.01 (0.997, 1.03)	22–28	1.02 (1.00, 1.03) *

Note: * *p* < 0.05; Medicaid, medical care; NHI, national health insurance.

**Table 4:** Relative risk of subarachnoid hemorrhage with every 1 °C change in hot and cold temperatures by climate zone.

Temperate Zone	HDDs (Cold Period) (Threshold: −3.5 °C by Mean Temperature)		CDDs (Hot Period) (Threshold: 31.5 °C by Maximum Temperature)	
	Lag	RR (95% CI)	Lag	RR (95% CI)
	0	1.22 (1.10, 1.35) *^,a^	1	1.08 (1.02, 1.14) *^,b^
Cold zone ^c^	2–3	1.01 (1.00, 1.02) *	-	-
Hot zone ^d^	-	-	4–7	1.03 (1.00, 1.06) *

Note: * *p* < 0.05; SAH, subarachnoid hemorrhage; HDDs, heating degree days; CDDs, cooling degree days; ^a^ Temperate zone with low cooling degree days (IQR, interquartile range; Q4 fourth quartile value); ^b^ Hot zone with high cooling degree days (IQR; Q1, first quartile value); ^c^ Temperate zone with lower heating degree days (IQR; Q4); ^d^ Cold zone with high heating degree days (IQR; Q1).

## Supplementary Materials

The following are available online at www.mdpi.com/1660-4601/14/4/449/s1, Table S1: Characteristics of daily meteorological variables and air pollutants in temperature zones by season (2004–2012), Table S2: Number of admissions for subarachnoid hemorrhage and monthly average temperature in temperate zone and hot zone, Table S3: Number of admissions for subarachnoid hemorrhage and monthly average temperature in temperate zone and cold zone.
