# Supplementary material for: Associations between Temperature and Hospital Admissions for Subarachnoid Hemorrhage in Korea
Source: Int J Environ Res Public Health. 2017 Apr 21;14(4):449. doi: 10.3390/ijerph14040449 (PMC5409649; doi:10.3390/ijerph14040449)
Supplement: Supplementary file 1 [file ijerph-14-00449-s001.pdf]

**Table S1.** Characteristics of daily meteorological variables and air pollutants in temperature zones by season (2004–2012)

| Parameter, Mean (SD) | CDDs | | | | | | | | HDDs | | | | | | | |
|---|---|---|---|---|---|---|---|---|---|---|---|---|---|---|---|---|
| | **Spring** | | **Summer** | | **Autumn** | | **Winter** | | **Spring** | | **Summer** | | **Autumn** | | **Winter** | |
| | **Temperate [a]** | **Hot [b]** | **Temperate [a]** | **Hot [b]** | **Temperate [a]** | **Hot [b]** | **Temperate [a]** | **Hot [b]** | **Temperate [c]** | **Cold [d]** | **Temperate [c]** | **Cold [d]** | **Temperate [c]** | **Cold [d]** | **Temperate [c]** | **Cold [d]** |
| **Heat Wave and Cold Spell** | | | | | | | | | | | | | | | | |
| Number of hot[e] /cold[f] days | - | - | 13.07 (9.51) | 26.92 (9.14) | - | - | 8.38 (10.92) | 0.83 (1.80) | - | - | 18.82 (12.41) | 15.39 (8.31) | - | - | 0.12 (0.41) | 9.28 (9.63) |
| Hot[g] /cold[h] duration (days) | - | - | 2.68 (3.23) | 3.65 (4.3) | - | - | 2.35 (2.22) | 1.5 (0.79) | - | - | 3.24 (3.8) | 2.66 (3.06) | - | - | 1.38 (0.49) | 2.23 (1.94) |
| **Other Meteorological Variables** | | | | | | | | | | | | | | | | |
| Precipitation (mm) | 2.57 (8.67) | 2.69 (8.82) | 9.05 (24.25) | 8.14 (21.92) | 2.9 (11.23) | 2.69 (11.66) | 0.78 (3.54) | 1.11 (4.32) | 3.4 (10.97) | 2.45 (8.07) | 7.84 (22.74) | 10.2 (26.57) | 2.82 (12.04) | 2.97 (12.06) | 1.18 (5.15) | 0.72 (3.13) |
| Precipitation (log mm) | –0.06 (1.84) | –0.04 (1.84) | 0.4 (2.21) | 0.24 (2.36) | –0.01 (1.73) | –0.08 (1.78) | –0.5 (1.92) | –0.43 (1.96) | 0.03 (1.88) | –0.15 (1.96) | 0.24 (2.28) | 0.34 (2.43) | –0.07 (1.77) | –0.07 (1.83) | –0.32 (1.82) | –0.53 (1.92) |
| Relative humidity (%) | 61.37 (15.41) | 57.17 (16.04) | 78.21 (10.98) | 73.58 (11.03) | 69.51 (12.9) | 66.1 (12.55) | 59.68 (15.12) | 57.12 (15.95) | 58.65 (17.01) | 58.27 (14.85) | 75.79 (11.1) | 73.98 (11.85) | 64.17 (13.76) | 67.43 (12.79) | 52.37 (17) | 59.72 (14.26) |
| Sea-level pressure (hPa) | 1014.87 (6.33) | 1015.07 (6.17) | 1007.64 (4.29) | 1007.9 (4.19) | 1018.18 (5.59) | 1018.05 (5.56) | 1023.73 (5.21) | 1023.37 (5.03) | 1014.97 (6.09) | 1014.86 (6.33) | 1008.08 (4.16) | 1007.49 (4.25) | 1017.74 (5.5) | 1018.36 (5.61) | 1022.76 (4.97) | 1024.16 (5.17) |
| **Level of Air Pollutants** | | | | | | | | | | | | | | | | |
| PM10 (μg/m$^3$) | 66.59 (46.36) | 62.34 (44.37) | 43.2 (22.74) | 41.19 (19.78) | 48.47 (26.63) | 45.41 (23.69) | 59.55 (30.23) | 52.19 (25.33) | 61.52 (45.56) | 67.39 (46.9) | 42.53 (19.3) | 43.16 (24.38) | 44.68 (22.53) | 48.08 (27.18) | 49.76 (23.16) | 61.7 (30.53) |
| $NO_2$ (ppm) | 0.02 (0.01) | 0.02 (0.01) | 0.02 (0.01) | 0.01 (0.01) | 0.02 (0.01) | 0.02 (0.01) | 0.03 (0.01) | 0.03 (0.01) | 0.02 (0.01) | 0.03 (0.01) | 0.02 (0.01) | 0.02 (0.01) | 0.02 (0.01) | 0.03 (0.01) | 0.02 (0.01) | 0.03 (0.01) |

SD, Standard deviation; CDDs, cooling degree days; HDDs, heating degree days; [a] Temperate zone with low cooling degree days (IQR; Q4, fourth quartile value); [b] Hot zone with high cooling degree days (IQR; Q1, first quartile value); [c] Temperate zone with low heating degree days (IQR; Q4); [d] Cold zone with high heating degree days (IQR; Q1); [e] Number of days with an heat index (HI) above 41 °C in summer; [f] Number of days with a minimum temperature below –12 °C in winter; [g] Consecutive days with an HI above 41 °C in summer; [h] Consecutive days with a minimum temperature below –12 °C in winter.

**Table S2.** Number of admissions for subarachnoid hemorrhage and monthly average temperature in temperate zone and hot zone

| Month | Number of Admissions | | Temperature | | | | | |
|---|---|---|---|---|---|---|---|---|
| | | | Mean [a] | | Max [b] | | DTR | |
| | Temperate [c] | Hot [d] | Temperate [d] | Hot [d] | Temperate [c] | Hot [d] | Temperate [c] | Hot [d] |
| January | 1973 | 2321 | −1.90 | 0.78 | 3.07 | 5.64 | 9.36 | 8.96 |
| February | 1897 | 2153 | 0.97 | 3.35 | 6.22 | 8.64 | 9.92 | 9.90 |
| March | 2084 | 2174 | 5.43 | 7.47 | 10.70 | 13.12 | 10.06 | 10.69 |
| April | 1975 | 2054 | 11.52 | 13.42 | 17.26 | 19.60 | 10.94 | 11.79 |
| May | 2001 | 2131 | 17.07 | 18.75 | 22.61 | 24.61 | 10.34 | 10.99 |
| June | 1833 | 2034 | 21.36 | 22.77 | 26.30 | 27.88 | 9.03 | 9.33 |
| July | 1932 | 2155 | 24.06 | 25.79 | 27.80 | 29.94 | 6.54 | 7.30 |
| August | 1914 | 1954 | 25.21 | 26.58 | 29.40 | 30.98 | 7.33 | 7.73 |
| September | 1832 | 1993 | 20.75 | 22.21 | 25.57 | 26.94 | 8.64 | 8.49 |
| October | 2093 | 2139 | 14.76 | 16.43 | 20.66 | 22.32 | 10.69 | 10.75 |
| November | 2081 | 2206 | 7.81 | 9.77 | 12.98 | 15.28 | 9.71 | 10.19 |
| December | 2311 | 2523 | 0.17 | 2.72 | 4.87 | 7.63 | 8.91 | 9.10 |
| Total | 23,926 | 25,837 | 12.32 | 14.22 | 17.33 | 19.43 | 9.28 | 9.6 |

DTR, diurnal temperature change; [a] Mean temperature; [b] Maximum temperature; [c] Temperate zone with low cooling degree days (IQR; Q4); [d] Hot zone with high cooling degree days (IQR; Q1)

**Table S3.** Number of admissions for subarachnoid hemorrhage and monthly average temperature in temperate zone and cold zone

| Month | Number of Admissions | | Temperature | | | | | |
|---|---|---|---|---|---|---|---|---|
| | | | Mean [a] | | Min [b] | | DTR | |
| | Temperate [c] | Cold [d] | Temperate [c] | Cold [d] | Temperate [c] | Cold [d] | Temperate [c] | Cold [d] |
| January | 3001 | 4225 | 2.04 | –2.85 | –1.60 | –7.25 | 8.12 | 9.32 |
| February | 2860 | 3813 | 4.38 | 0.36 | 0.31 | -4.35 | 8.86 | 10.01 |
| March | 2925 | 4296 | 8.03 | 5.10 | 3.71 | 0.21 | 9.32 | 10.29 |
| April | 2780 | 3943 | 13.48 | 11.69 | 8.91 | 6.17 | 9.84 | 11.54 |
| May | 2865 | 4077 | 18.27 | 17.73 | 14.14 | 12.47 | 9.10 | 11.10 |
| June | 2807 | 3900 | 21.98 | 22.30 | 18.55 | 17.83 | 7.72 | 9.74 |
| July | 2967 | 3969 | 25.17 | 24.66 | 22.44 | 21.62 | 6.33 | 6.93 |
| August | 2587 | 3757 | 26.44 | 25.75 | 23.59 | 22.37 | 6.67 | 7.75 |
| September | 2666 | 3896 | 22.48 | 21.00 | 19.32 | 16.90 | 7.26 | 9.14 |
| October | 2821 | 4178 | 17.35 | 14.55 | 13.32 | 9.51 | 9.09 | 11.17 |
| November | 2826 | 4141 | 10.90 | 7.17 | 6.88 | 2.57 | 8.86 | 9.87 |
| December | 3259 | 4762 | 4.09 | –0.86 | 0.29 | –5.05 | 8.29 | 8.91 |
| Total | 34,364 | 48,957 | 14.6 | 12.27 | 10.87 | 7.8 | 8.28 | 9.64 |

DTR, diurnal temperature change; [a] Mean temperature; [b] Maximum temperature; [c] Temperate zone with low cooling degree days (IQR; Q4); [d] Hot zone with high cooling degree days (IQR; Q1)
